# Physical, Cognitive and Social Rehabilitation in Relation to Sleep Quality and Cognitive Functions in the Elderly

**DOI:** 10.3390/ijerph18105148

**Published:** 2021-05-13

**Authors:** Karolina Filipczyk, Joanna Smolarczyk-Kosowska, Łukasz Kunert, Przemysław Filipczyk, Paweł Dębski, Magdalena Piegza, Robert Pudlo

**Affiliations:** 1 Department of Psychiatry, Faculty of Medical Sciences in Zabrze, Medical University of Silesia, 42-612 Katowice, Poland; joanna.smolarczyk@med.sum.edu.pl (J.S.-K.); lkunert@sum.edu.pl (Ł.K.); pdebski@sum.edu.pl (P.D.); mpiegza@sum.edu.pl (M.P.); rpudlo@sum.edu.pl (R.P.); 2 Faculty of Health Sciences, Jan Długosz University in Czestochowa, 42-200 Czestochowa, Poland; filipmail@o2.pl

**Keywords:** physical activity, older adults, cognitive function, active aging, mental health

## Abstract

The aim of the study was to assess cognitive functions and sleep quality after a 3-month holistic intervention including physical, social and cognitive rehabilitation in patients 65+. Twenty-nine people participated in the study. The study was divided into two stages. In the first stage, a self-administered questionnaire consisting of sociodemographic questions was used, and cognitive functions were assessed using the Rey-Osterrieth complex figure test, Addenbrooke’s Cognitive Examination III (ACE III) test, Montreal Cognitive Function Assessment Scale (MoCA) and digit repetition test. All patients were also assessed for sleep quality using the Athens Insomnia Scale (AIS). After three months, the patients were assessed for cognitive functions and sleep quality, which was the second stage of the study. Analysis of the results obtained by patients in the study showed a statistically significant improvement in sleep quality and cognitive function. Rehabilitation activities, including physical training, cognitive exercises and occupational therapy, reduce the severity of mild cognitive disorders and reduce insomnia.

## 1. Introduction

As the global population ages, cognitive impairment associated with longevity and deterioration of sleep quality is becoming an increasingly serious global problem, very often leading to insomnia. Memory impairment is one of the most frequently reported complaints by the elderly at the general practice (GP) surgeries. A medical condition called a mild cognitive impairment (MCI) is a common condition in the elderly. It is characterized by deterioration of memory, attention, and cognitive function that is beyond what is expected based on age and educational level [1]. Individuals with MCI report impairment of memory or other cognitive functions, but still function independently and their daily life activity is maintained, although minor disorders may occur in this area. Deterioration of cognitive functioning should be confirmed by an objective interview and tests assessing cognitive functional ability. Severity of neurocognitive impairment in MCI is greater than in age-related memory impairment and, unlike subjective cognitive impairment, it is objectively confirmed [2,3]. Worldwide estimated costs associated with premature dementia and, in consequence, inability to work and long-term sick leave outweigh the costs arising from chronic diseases such as diabetes [4]. Alzheimer’s disease—the main cause of dementia—has reached the size of a pandemic [4], and it constitutes not only an economic, but also a social problem, especially because it is safe to say that the problem does not concern one person, but the entire family involved in caring for the patient. Increasing insomnia can lead to deterioration of cognitive function. Even up to 70% of older people who go to their GP experience symptoms of insomnia [5]. Insomnia is linked to the socio-economic status, racial and ethnic classification, family relationships, general health and mental condition, as well as neurocognitive functioning and subsequent dementia [6,7].

Research conducted in Finland shows how the early impact of geriatric intervention affects the prevention of cognitive impairment and disability. FINGER (The Finnish Geriatric Intervention Study to Prevent Cognitive Impairment and Disability) project is at the forefront of international efforts to address public health clinical problems related to the early identification of people at increased risk of cognitive impairment late in life, and it is developing intervention strategies to prevent or delay the onset of cognitive impairment and dementia [8]. The FINGER project involved people aged 60–77. The study group consisted of 631 patients and the control group of 629. These studies show that the use of FINGER intervention is not significantly limited by socio-economic status, cognitive abilities or cardiovascular risk level [8]. Is an example of modern psychogeriatric interventions. The results of this large, long-term, randomized, controlled trial suggest that multi-domain intervention may improve or maintain cognitive function in at-risk elderly people in the general population [9].

Modern psychiatry meets the needs of civilization, focusing on environmental psychiatry. Community care is a model that is more responsive to the patient‘s needs and is also more effective in terms of the use of financial resources and therapeutic effectiveness [10]. The World Psychiatric Association (WPA) defines environmental psychiatry through various practices and concepts, such as meeting the needs of the population in the most accessible way, promoting a wide support network, services and resources and also emphasizes the importance of maintaining and supporting the ability of patients to perform social roles relevant to healthy people [11].

In 2019, the Support Centre 65+ (CW65+) was established in Tarnowskie Góry. A network of health-promoting activities has been implemented, aimed at maintaining, restoring and improving mental health, improving cognitive functions and sleep quality. CW65+ patients participated in the three-month long proprietary rehabilitation program created in response to the needs of the participants.

So far, we did not have a psychogeriatric ward and community treatment team in the district which resulted in the extension of the diagnostic process and, consequently, more frequent and longer psychiatric hospitalizations. Creating opportunities for out-of-hospital care increases patients’ sense of security without distracting them from their home environment. In March 2020, CW65+ was discontinued due to the COVID-19 outbreak.

The aim of the study was to assess cognitive functions and sleep quality after a 3-month holistic intervention including physical, social and cognitive rehabilitation in patients 65 years and over.

Guided by the prevailing health complaints, an attempt was made to test the hypothesis that coordinated rehabilitation interventions delivered in an outpatient setting improve cognitive function and reduce sleep disturbances.

## 2. Materials and Methods

The study group consisted of participants of the project named: “Support Centre 65+ in Tarnowskie Góry”. In order to ensure a naturalistic course of the study, the maximum liberal criteria of inclusion were established: the required age (65 years and over) and place of residence in the Tarnowskie Góry, declared cognitive impairment, difficulties in daily functioning, general health condition allowing safe use of the offered activities and the possibility of giving informed consent for participation in the project. The plan for the study was to include all project participants, i.e., four, 16-person groups, which proved impossible due to the outbreak of the COVID-19 pandemic, so only the first two groups of patients, whose stay at the Center included the period from August 2019 to March 2020, were finally studied. Exclusion criteria were: addiction to alcohol or other psychoactive substances with the inability to maintain 3 months of abstinence, withdrawal of consent to participate in the project or deterioration of health status preventing further use of rehabilitation activities, and suspicion of depression in the screening.

The presence of depression in the patient, confirmed by psychiatric examination and the necessity to implement of pharmacotherapy connected with it excluded the patient from participation in the study, but not from the project.

The study, apart from the original questionnaire consisting of sociodemographic questions, was carried out in two time points: before the beginning of the intervention and after 3 months of stay, using: for the study of cognitive functions: Rey-Osterrieth’s compound figure, Addenbrooke’s Cognitive Examination III (ACE III) test, Montreal Cognitive Function Assessment Scale (MoCA), digit repetition test. All patients were also evaluated for sleep quality using the Athens Insomnia Scale (AIS). The tests were always performed in the morning between 9 and 11 am, during the first and last week of participation.

The qualification of participants for rehabilitation was based on specific elements of the Fullerton test, considered particularly useful in the multidimensional assessment of the physical fitness of elderly people (6 min walking test (6MWT), with the assessment of the fatigue level according to the modified Borg scale, Back Scratch Test, Up and Go Test, Sit-and-Reach Test). During the qualification, patients were also subjected to a Romberg test to assess possible imbalance and gait disorders. The patients’ physical fitness was also assessed regularly, in weeks 6 and 12 of their stay in CW65+. The rehabilitation plan was set individually on the basis of the initial test results and the patients’ expectations. The treatment program included group exercises improving physical fitness, breathing and relaxation exercises with elements of body awareness exercises. Blood pressure and blood glucose levels were measured daily.

Within the rehabilitation program, patients performed physical exercises taking into account, inter alia: breathing exercises, cardiovascular exercises, isometric exercises, stabilization exercises and stretching exercises. The program was arranged in such a way that every senior could easily perform the exercise after prior instruction and demonstration by a qualified physiotherapist. A single meeting consisted of standard stages such as warming up, the main part as well as calming down and calming down the body. Exercise meeting took 30 min each time. At each meeting, exercises were performed that enabled the involvement of many muscle groups, with particular emphasis on those areas that are most often weakened in the senior group, such as core stabilization, the gluteal muscle group, the thigh muscles and other spine extensors.

Patients benefited from art therapy, horticultural therapy, dance movement therapy, music therapy, culinary training, passive bibliotherapy, activity-based therapy and cognitive training using computer programs (Neuroforma, CogniPlus, RehaCom), psychological workshops, group classes, individual meetings with a psychologist and psychiatric and internal medicine consultations. The intensity of physical exercise and cognitive training depended on the patient’s initial condition and predisposition, which were assessed individually by a physiotherapist, psychiatrist and an internal medicine specialist.

The Bioethics Committee of the Medical University of Silesia decided that the study entitled “Physical, cognitive and social rehabilitation in relation to sleep quality and cognitive functions in the elderly” does not require the consent of this committee (Decision number PCN/0022/KB/179/19).

### Description of the Used Diagnostic Tools

Cognitive disorders may concern various levels of information processing, ranging from stimulus perception, through the level of working memory, attention deficits and executive functions. The study of cognitive functions is prognostic because it determines the level of cognitive functioning, and thus also of the psychosocial functioning of the patient. The following methods were used in the study:

1. The Rey-Osterrieth complex figure test is used to test the level of perceptual structuring, attention and visual-motor control, and direct visual memory. This is a paper-pencil test, it can be used in individual and group tests. The figure itself has no meaning; it does not resemble any real object. Eighteen simple and complex elements can be distinguished in it. The maximum score that can be obtained is 36 points. It consists of three test conditions: copy, immediate recall and delayed recall. At the first step, subjects are given the test stimulus card, and then asked to draw the same figure. Subsequently, they are instructed to draw what they remembered [12]. Subjects were asked to perform in pencil as much as possible the most exact copy of Rey’s figure on a blank sheet of paper A4 format. The Polish instruction does not specify the requirements regarding the time interval between tests.
2. The digit repetition subtest from the Wechsler Adult Intelligence Scale WAIS-R(PL) is used to examine structural and processual parameters of working memory. The repeating digits straight subtest measures working memory capacity, whereas the repeating digits clock procedure measures information processing efficiency. The overall test score is the sum of the scores on both scales (repeating digits straight and repeating digits backwards). The maximum score is 28 points and the minimum score is 0 points [13]. The manual does not specify a test interval requirement.
3. The Montreal Cognitive Assessment Scale (MoCA), designed by Z. Nasreddine, is a screening tool for detection of mild cognitive impairment (MCI). This tool is used to evaluate cognitive functions such as short-term memory, visualspatial, executive and linguistic functions, verbal fluency, attention, naming, abstraction and allopsychic orientation [10]. The maximum number of points is considered to be normal cognitive functioning. Moreover, the MoCA scale as a screening tool allows the assessment of a greater number of cognitive functions than, for example, the commonly used Mini Mental State Evaluation (MMSE) studies [14,15]. Months, an alternative version of the questionnaire was used, which can be performed one month after the first study.
4. Addenbrooke’s Cognitive Examination III (ACE III) is an enhanced cognitive screening tool useful in early detection of cognitive disorders, initial differential diagnosis of dementia syndromes and monitoring of disease progression. ACE III evaluates attention and orientation, memory, verbal fluency, language and visualspatial functions. Is considered a comprehensive screening tool for cognitive impairment. The scale can be used by doctors and psychologists, both as a screening tool and as an introduction to a comprehensive neuropsychological assessment [16]. The maximum score that can be obtained is 100 points. The Polish instruction does not specify the requirements regarding the time interval between tests. It has a parallel version [17].
5. All patients were also assessed for sleep quality using the Athens Insomnia Scale (AIS). It is the first tool making it possible to assess the symptoms associated with insomnia, which has Polish validation. The studies confirmed good psychometric properties of the scale. It consists of eight items for falling asleep, waking up at night, waking up in the morning, total sleep time, sleep quality, well-being the next day, mental and physical fitness the next day, and daytime sleepiness. Each ingredient is rated on a score scale from 0 (no difficulty) to 3 (severe difficulty). The total AIS score of 8 or more points was considered to be the value, which makes it highly possible to infer inorganic insomnia according to the ICD-10 criteria. The conciseness, reliability and relevance of AIS make this tool useful in clinical practice and scientific research [18].

The collected data was analyzed with the use of Excel 2016 and Statistica version 13.3 (TIBCO Software Inc., Palo Alto, CA, USA). The assessment of the normality of distributions was conducted with the use of the Shapiro–Wilk test. The distributions were different from normal. The relationships between quantitative variables were evaluated with the Spearman’s rank correlation coefficient. The Chi-square compliance test was applied for the nominal variables. The Wilcoxon test was used to compare the results obtained by the patients in the first and second testing. Gender differences were assessed with the use of the Mann–Whitney U test. A significance level of *p* < 0.05 was adopted in the statistical procedures.

## 3. Results

The study involved 32 patients of the Support Centre 65+ in Tarnowskie Góry, 3 people were excluded. One person was excluded from the study because they were blind, which prevented them from completing the tests, the second person obtained a score of 25 on the Beck depression scale, which may indicate symptoms of depression and this could affect the quality of the obtained results, the third person withdrew consent during the study. The program aimed to carry out the study among 64 people, unfortunately in March 2020 the program was suspended due to the outbreak of COVID-19. Among the respondents there were 18 women and 11 men. The program was dedicated to seniors in need of mental health support. Among the respondents, two persons admitted to be compulsive smokers, 26 persons were treated for hypertension, eight persons for diabetes II, four persons for coronary heart disease. Of the 18 women, 12 were widows and of the 11 men, and eight were widowers. In all patients participating in the study, there was no need to modify their current treatment during the course of the project. A description of the study group is shown in Table 1.

**Figure 1:**
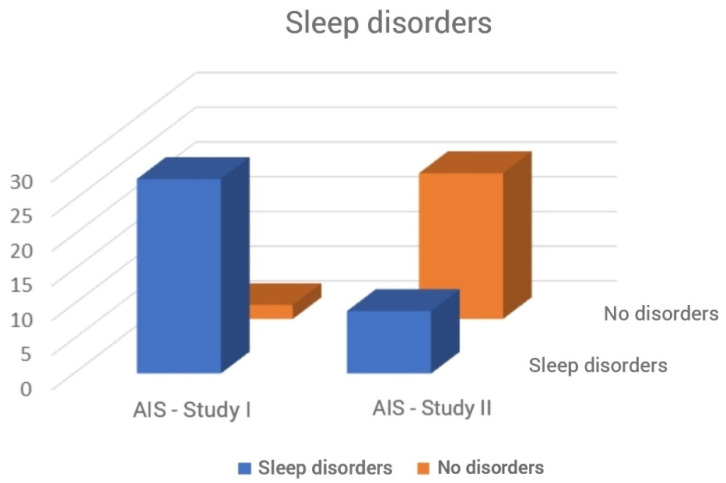
Frequency of sleep disorders (Chi-square test). AIS, Athens Insomnia Scale. Chi2 = 25.45; *p* < 0.001.

**Figure 2:**
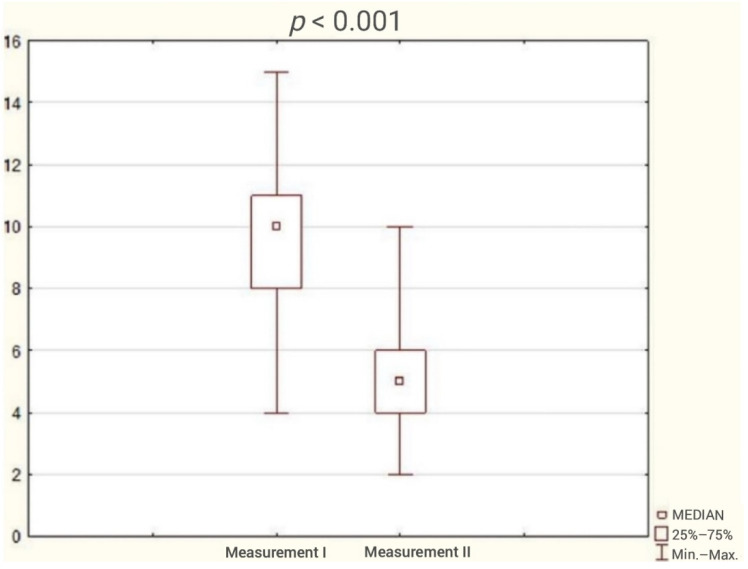
Patient result in Athens Insomnia Scale (AIS) during the first and second study (Wilcoxon test).

**Figure 3:**
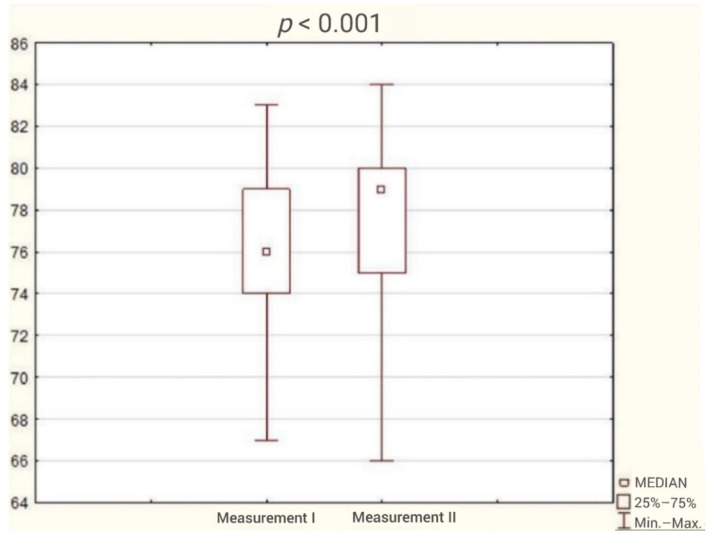
Patient result in Addenbrooke’s Cognitive Examination III (ACE III) during the first and second study (Wilcoxon test).

**Figure 4:**
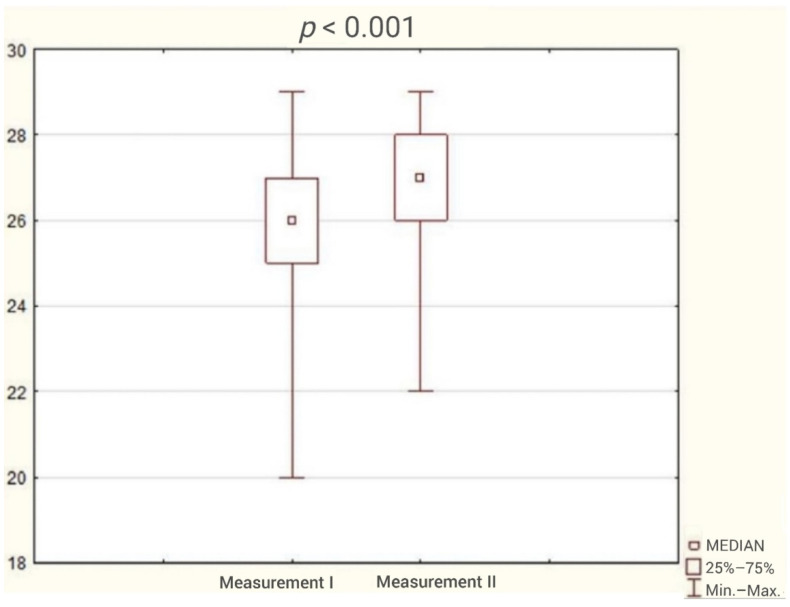
Patient result in Montreal Cognitive Assessment Scale (MoCA) during the first and second study (Wilcoxon test).

**Figure 5:**
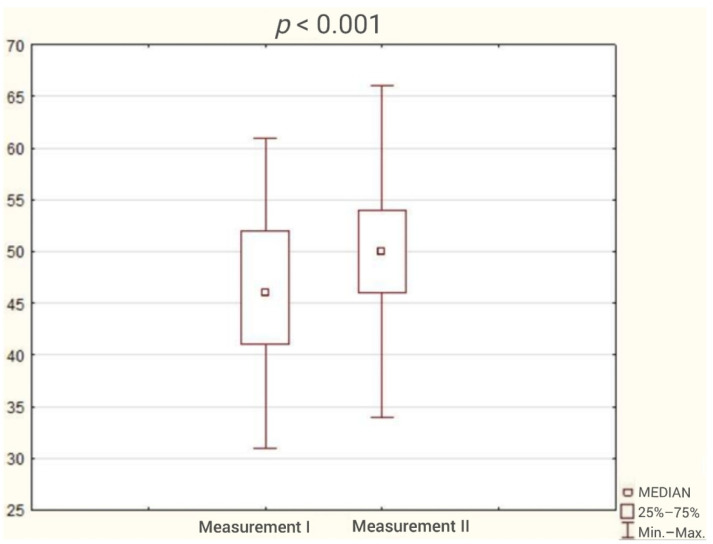
Patient result in Rey’s complex figure test during the first and second study (Wilcoxon test).

**Figure 6:**
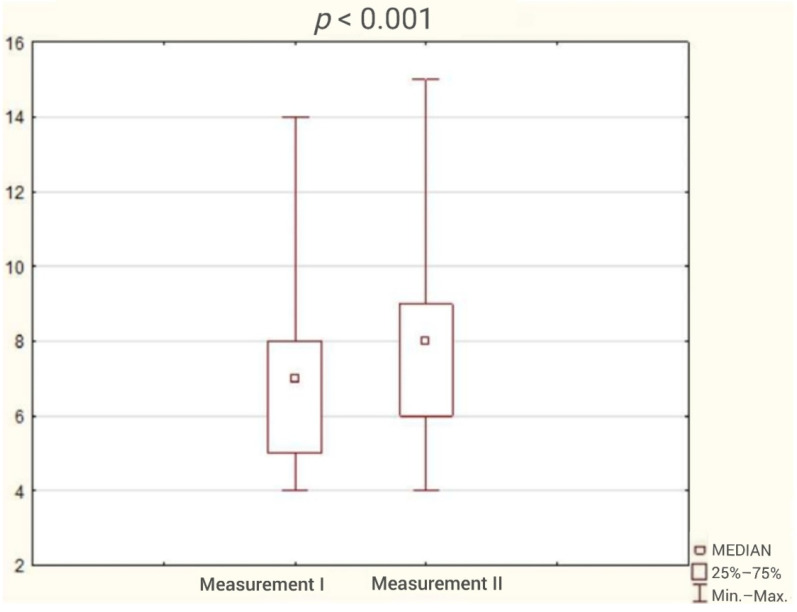
Patient result in digit repetition test during the first and second study (Wilcoxon test).

The average duration of participation in the program was 3 months. The analysis of the results obtained by patients in the questionnaires showed reduction in the incidence of sleep disorders (Figure 1) and a statistically significant improvement in sleep quality and cognitive function. The test results are presented in Table 2 and Figure 2, Figure 3, Figure 4, Figure 5 and Figure 6.

No correlation was proved between sleep disorders and cognitive impairment neither in the first and second study (Spearman’s rank correlation test; *p* > 0.05).

There was no significant static difference between the incidence and severity of sleep disorders by gender (Chi-square test; Mann–Whitney’s test; *p* > 0.05).

## 4. Discussion

The results of the presented study confirmed the hypothesis that active participation in the rehabilitation program in the 65+ Support Centre significantly improved the quality of sleep and cognitive functioning.

In the patients included in the program was observed high level of motivation to work with a psychologist, physiotherapist, dietician and doctor. The aim of the study conducted at the 65+ Support Center was to assess the impact of daily physical exercise, psychoeducation including nutritional tips, cognitive exercises and developing social activity on the quality of sleep as well as their role in preventing or delaying cognitive deterioration. Our analysis shows that thanks to properly selected methods of rehabilitation, staying in a group of people and psychoeducation, it is possible to significantly improve the quality of sleep and cognitive functions. The aging process is associated with an increased incidence of sleep related ailments [19,20]. Elderly people have problems with falling asleep and maintaining night sleep continuity [19]. Changes in sleep patterns are part of the normal aging process, so both elderly people and their caregivers should be educated in this regard. Awareness raising programs are extremely important and helpful in recognizing sleep disorders and coping with sleep problems of elderly people. Ageing does not always mean poor sleep quality, but good sleep can certainly improve the overall health condition [19,20]. Insomnia is a global problem. Its prevalence among the elderly is constantly growing. The Chongqing study showed a significant correlation of insomnia with coronary artery disease, dizziness, chronic pain, anorexia, malnutrition, depression, cognitive impairment [21]. Both this study and the primary care study in India showed that women are more at risk of insomnia than men [21,22]. This relationship is confirmed by most of the analyzes described so far [23], with the exception of a study conducted in Korea, the results of which suggest that the quality of marriage is the most important factor [24]. In our study, there was no statistically significant difference between the prevalence and severity of sleep disorders between men and women, which may be due to the fact that most of the seniors studied were single (widow/widower) or the small size of the study group. Numerous studies show that both physical exercise and cognitive activity can significantly reduce the risk of sleep disorders in elderly people [25,26,27]. At the same time, sleep disorders can cause cognitive impairment, although in elderly people this relationship is less pronounced than in young people [28]. In our study it was not observed, significant relationship between the severity of sleep disorders and the occurrence of cognitive impairment was observed, the reason for this may be the insufficient size of the study group. Attention should be drawn to the fact that cognitive disorders not only disorganize the life of the patient and their caregivers, but also affect the increase in possible falls and postural stability disorders. The available literature shows that, there is a high correlation between cognitive disorders and the physiological profile among elderly people. A study conducted by the University of Humanities and Life Sciences in Częstochowa shows that the higher the cognitive deficit, the greater the risk of a fall in this group [29]. Properly selected rehabilitation can prolong the independent functioning of elderly people and improve their daily functioning, maintaining an appropriate balance. Numerous multicentre studies of the population of elderly people from different cultural circles confirmed the fact that active, healthy lifestyle, associated with physical effort and appropriate degree of nutrition, is a condition of good mental and intellectual health, being the most important factor for successful aging [30,31]. Most studies confirm the relationship between the level of physical activity and the occurrence of mild cognitive disorders [32]. Physical activity, as one of the methods that can delay the conversion of MCI to dementia, is a low-risk method, provided, of course, that the type of physical effort is adapted to a specific patient (his age, lifestyle, physical condition and somatic state). It is also a cheap and widely available method [33], which we have also used it successfully in our patients. Based on the results of studies conducted in people over the age of 65, it has been proven that regular and prolonged physical activity is associated with a higher level of cognitive functions, and later in life with a lower risk of cognitive disorders, the so-called successful aging [34], we can conclude that physical activity should be promoted as a modifiable preventive factor contributing to the improvement of well-being and increasing the efficiency of cognitive functions [35].

Based on the FINGER project, we can conclude that physical exercise, appropriately selected rehabilitation, improve cognitive functions in old age, therefore we can treat psychogeriatric wards as a kind of prevention or at least delaying the onset of dementia symptoms.

Creating facilities such as the 65+ Support Centre in Tarnowskie Góry allows seniors to receive comprehensive medical and psychological care and creates conditions for maintaining an adequate level of physical and social activity, which translates into improving many aspects of their daily functioning. In conclusion, despite the small group, the results of the study seem to be promising. After three months, all respondents showed an improvement in cognitive functions and a reduction in insomnia.

### Study Limitations

The most serious limitation of our study is the small size of the study group, resulting from the need to suspend the qualification of participants for the Program and the activities of the 65+ Support Centre due to epidemiological threats during the COVID-19 pandemic. Another limitation is the lack of a control group and no objective measures of sleep. The last limitation is also the possibility of a priming error when re-using the cognitive test tools, which we tried to eliminate with the help of alternative versions of the questionnaires.

## 5. Conclusions

Rehabilitation classes, including physical training, cognitive exercises and occupational therapy, reduce the severity of mild cognitive disorders and they reduce insomnia.

## Figures and Tables

**Table 1:** Sociodemographic data relating to the study group.

Characteristics of the Study Group	Number of Respondents
Sex	
Male, *n* (%)	11 (37.9%)
Female, *n* (%)	18 (62.1%)
Age	
Minimum, years	65
Maximum, years	92
Median, years	78.5
Education	
Primary, *n* (%)	3 (10.4%)
Vocational and secondary, *n* (%)	19 (65.5%)
Higher, *n* (%)	7 (24.1%)
Housing situation	
Living alone, *n* (%)	18 (62.1%)
Living with family, *n* (%)	11 (37.9%)
Matrimonial status	
Widow/Widower, *n* (%)	20 (68.9%)
Married, *n* (%)	9 (31.1%)
Somatic diseases	
Hypertension, *n* (%)	26 (89.6%)
Diabetes mellitus t.II, *n* (%)	8 (27.5%)
Coronary heart disease, *n* (%)	4 (13.7%)
Smoking cigarettes	
Yes, *n* (%)	2 (6.8%)
No, *n* (%)	27 (93.2%)

**Table 2:** Results achieved by patient on the scales during the first and second studies (Wilcoxon test).

	Study I			Study II				
	Median	Q1	Q3	Median	Q1	Q3	Z	*p*
Athens Insomnia Scale	10	8	11	5	4	6	4.54	<0.001
Addenbrooke’s Cognitive Examination III	76	74	79	79	75	80	4.38	<0.001
Montreal Cognitive Assessment Scale	26	25	27	27	26	28	3.82	<0.001
Rey-Osterrieth complex figure test	46	41	52	50	46	54	4.27	<0.001
Digit repetition test	7	5	8	8	6	9	3.82	<0.001

## Data Availability

The data that support the findings of this study are available from the corresponding author, upon reasonable request.
